# 2'-*O*-methylation of the mRNA cap protects RNAs from decapping and degradation by DXO

**DOI:** 10.1371/journal.pone.0193804

**Published:** 2018-03-30

**Authors:** Frédéric Picard-Jean, Carolin Brand, Maude Tremblay-Létourneau, Andréa Allaire, Maxime C. Beaudoin, Simon Boudreault, Cyntia Duval, Julien Rainville-Sirois, Francis Robert, Jerry Pelletier, Brian J. Geiss, Martin Bisaillon

**Affiliations:** 1 Département de Biochimie, Faculté de Médecine et des Sciences de la Santé, Université de Sherbrooke, Sherbrooke, QC, Canada; 2 Department of Biochemistry, McGill University, Montréal, QC, Canada; 3 Department of Microbiology, Immunology and Pathology, Colorado State University, Fort Collins, CO, United States of America; Korea University, REPUBLIC OF KOREA

## Abstract

The 5' RNA cap structure (^m7^GpppRNA) is a key feature of eukaryotic mRNAs with important roles in stability, splicing, polyadenylation, mRNA export, and translation. Higher eukaryotes can further modify this minimal cap structure with the addition of a methyl group on the ribose 2'-*O* position of the first transcribed nucleotide (^m7^GpppN_m_pRNA) and sometimes on the adjoining nucleotide (^m7^GpppN_m_pN_m_pRNA). In higher eukaryotes, the DXO protein was previously shown to be responsible for both decapping and degradation of RNA transcripts harboring aberrant 5’ ends such as pRNA, pppRNA, GpppRNA, and surprisingly, ^m7^GpppRNA. It was proposed that the interaction of the cap binding complex with the methylated cap would prevent degradation of ^m7^GpppRNAs by DXO. However, the critical role of the 2’-*O*-methylation found in higher eukaryotic cap structures was not previously addressed. In the present study, we demonstrate that DXO possesses both decapping and exoribonuclease activities toward incompletely capped RNAs, only sparing RNAs with a 2’-*O*-methylated cap structure. Fluorescence spectroscopy assays also revealed that the presence of the 2’-*O*-methylation on the cap structure drastically reduces the affinity of DXO for RNA. Moreover, immunofluorescence and structure-function assays also revealed that a nuclear localisation signal is located in the amino-terminus region of DXO. Overall, these results are consistent with a quality control mechanism in which DXO degrades incompletely capped RNAs.

## Introduction

Eukaryotic pre-messenger RNAs (pre-mRNAs) must undergo several modifications to be protected from 5’-3’ exonucleases, transported from the nucleus to the cytoplasm, and translated. The first modification to occur is the addition of a guanosine nucleotide to the 5’ terminal nucleotide with a particular 5’-5’ linkage (capG). The inverted nucleotide is then methylated at the N7 position by an RNA guanine-N7 methyltransferase which forms the minimal cap structure (cap0) found in lower eukaryotes. The roles of the N7-methylation in stability, splicing, polyadenylation, mRNA export, and translation have been well defined and characterized for many years. For a recent review of mRNA capping and the biological roles of the cap0 structure see [1]. Higher eukaryotes can further modify this minimal cap structure with the addition of a methyl group on the ribose 2'-*O* position of the first transcribed nucleotide (cap1) and sometimes at the adjoining nucleotide (cap2) [2,3]. The function of the 2’-*O*-methylation remained elusive for several decades, and only recently it was shown to play a key role in the distinction between self and non-self RNAs [4,5]. Indeed, in higher eukaryotes, cellular mRNAs are 2’-*O*-methylated whereas non-self RNAs, such as certain viral RNAs, lack 2’-*O*-methylation. Moreover, non-2’-*O*-methylated RNAs can be detected and identified as foreign RNAs by cytoplasmic sensors such as MDA5 [5] and RIG-I [6,7], indicative of an infection. Activation of MDA5 and RIG-1 has been shown to induce type I interferon (INF) signaling, thereby promoting the expression of INF-stimulated genes (ISGs) whose protein products are involved in the innate immune response (reviewed in [8,9]). Since incompletely capped self RNAs could potentially be mistakenly recognized as extrinsic RNAs by cellular surveillance systems, cells must possess efficient mechanisms to prevent the activation of an undesired antiviral response.

The first evidence of a specialized quality control mechanism for the mRNA cap structure was demonstrated in lower eukaryotes. The Rai1 protein possesses a pyrophosphohydrolase (PPH) activity toward 5’-triphosphorylated RNA (pppRNA → PPi + pRNA) and a decapping activity toward unmethylated capped RNA (GpppNpRNA → GpppN + pRNA) [10,11]. Interestingly, the 5’-monophosphate RNA products resulting from Rai1 cleavages are substrates for its interacting partner Rat1 which harbors a 5’-3’ exoribonuclease activity [12,13]. It was previously proposed that Rai1 can sense and initiate the degradation of uncapped and incompletely capped RNAs which are then further degraded by Rat1 [10,11]. In yeast, the Dxo1 protein, which belongs to the same family as Rai1, was identified as another member of the quality control mechanism for the cap structure [14]. Both Rai1 and Dxo1 possess a similar decapping activity towards unmethylated cap structures, although Dxo1 shows measurable activity towards the methylated capped mRNA [11,14]. Furthermore, Dxo1 has no PPH activity but possesses a 5’-3’ exoribonuclease activity which allows this enzyme to solely clear transcripts with aberrant 5’ ends [14]. The discovery of the quality control mechanism for the synthesis of the cap structure shook the generally accepted notion that the addition of the cap structure always proceeded to completion. The deletion of Rai1 in yeast cells led to the accumulation of unmethylated transcripts upon nutrient starvation [11]. Additionally, when both Rai1 and Dxo1 were depleted, this phenotype occurred even under normal growth conditions [14].

In higher eukaryotes, pyrophosphohydrolase, decapping and 5’-3’ exoribonuclease activities are carried out by the DXO protein [15]. Such catalytic activities strongly suggest that DXO is an important constituent of the quality control mechanism that identifies and degrades improperly capped transcripts. Indeed, DXO previously showed hydrolysis activity towards transcripts with aberrant 5’ ends such as pRNA, pppRNA, GpppRNA (capG) and, surprisingly, ^m7^GpppRNA (cap0). The authors of the study suggested that this lack of specificity would most likely be thwarted in cells by the cap binding complex (CBC) which preferentially binds the methylated cap, thereby preventing degradation of cap0 RNAs by DXO [15]. In this scenario, the mammalian quality control mechanism would lead to the degradation of all incompletely capped mRNAs by DXO, independently of the nature of their 5’ end, unless they are initially bound by CBC.

In the present study, we reveal the unexpected importance of the 2'-*O*-methylation of the first transcribed nucleotide in the pre-mRNA 5' end capping quality control mechanism. Our studies indicate that both decapping and exoribonuclease activities of DXO are blocked by the 2'-*O*-methylation of the ribose moiety of the first nucleotide. This suggests that DXO spares fully capped (cap1) mRNAs while selectively degrading transcripts lacking the 2’-*O*-methylation (such as cap0 and capG).

## Materials and methods

### Cloning and protein expression

A plasmid for the expression of a full-length DXO protein (396 amino acids) was generated by inserting the human DXO gene (Open Biosystems, MHS1011-76814 Human MGC Verified FL cDNA) in the pET28a expression plasmid (Novagen). In this context, the DXO protein is fused in-frame with an N-terminal peptide containing six tandem histidine residues, and expression of the His6-tagged protein is driven by a T7 RNA polymerase promoter. The plasmid (pET28a-DXO) was transformed into *Escherichia coli* BL21(DE3) cells and 1L culture of *E*. *coli* BL21(DE3)/pET28a-DXO was grown at 37°C in LB medium containing 30μg/ml kanamycin until the OD_600_ reached 0.5. The culture was adjusted to 0.4mM isopropyl β-D-thiogalactoside (IPTG) and 2% ethanol, and the incubation continued at 18°C for 20h. The cells were then harvested by centrifugation at 3500g for 15min (Sorvall SLA-1500 rotor). All subsequent procedures were performed at 4°C. Bacteria pellets were resuspended in 30ml of buffer A (50mM Tris-HCl pH 7.5, 150mM NaCl and 10% sucrose), and cell lysis was achieved by adding lysozyme and Triton X-100 to final concentrations of 50μg/ml and 0.1%, respectively. The lysates were sonicated to reduce viscosity, and any insoluble material was removed by centrifugation at 12,000g for 45min at 4°C (Sorvall SS-34 rotor). The soluble extract was applied to a 2ml column of Protino-Ni-NTA agarose (Macherey-Nagel, #745400) that had been equilibrated with buffer A containing 0.1% Triton X-100. The column was washed with the same buffer supplemented with 5mM imidazole and then eluted stepwise with buffer B (50mM Tris-HCl pH 8.0, 100mM NaCl and 10% sucrose) containing 50mM, 100mM, 200mM, 500mM or 1000mM imidazole. The polypeptide composition of the eluate fractions was monitored by SDS-PAGE and western blotting using a penta-His antibody (Qiagen, #34660). The recombinant DXO protein was retained on the column and recovered in the 200mM and 500mM imidazole eluates. Following a 2h dialysis against buffer C (50mM Tris-HCl pH 8.0, 50mM NaCl, 2mM dithiothreitol (DTT) and 10% glycerol), the protein concentration was determined by a Bradford protein assay (Thermo Scientific, #23238) using BSA as standard.

### RNA synthesis

Two RNA substrates, ^5’^AGUAGUUCGCCUGUGUGAGCUGACAAACUUAGUAGUGUUUGUGAG^3’^ (45nt) and ^5’^AGUAGUUCGCCUGUGUGAGCUGACA^3’^ (25nt) for decapping assay and fluorescence spectroscopy assay, respectively, were produced using two complementary oligonucleotides with a 5’ overhang extended by 5 cycles of a 3 step PCR reaction. The resulting dsDNA, containing the T7 RNA promoter, was transcribed using recombinant T7 RNA polymerase in TRX buffer (80mM HEPES-KOH pH 7.5, 40mM DTT, 24mM MgCl_2_, 2mM spermidine) with 6.25mM rNTPs and 2mU Pyrophosphatase (Roche, #10108987001) during 3 hours at 37°C. Following transcription, the RNA substrate was purified on a denaturing polyacrylamide gel and visualized by UV-shadowing. The corresponding band was excised, and then eluted from the gel by an overnight incubation in a 0.1% SDS / 0.5M ammonium acetate solution. The eluate was precipitated using ethanol and 30mM sodium acetate pH 5.2, resuspended in nanopure water and quantitated by spectrophotometry. The purified 5’ triphosphorylated RNA was further processed with recombinant enzymes to add the different 5’ cap structures. RNA harbouring a 5’ capG was obtained by incubating 5μM recombinant human capping enzyme (HCE, expressed and purified as described in [16]) with 1000pmol of RNA, 5000pmol of [α-^32^P]GTP, and 10mU of Pyrophosphatase (Roche, #10108987001) in capping buffer (25mM Tris-HCl, 5mM MgCl_2_, 0.5mM DTT) for 2 hours at 37°C. Furthermore, 5’ cap0 RNA was synthesized using the purified recombinant N7-methyltransferase of *S*. *cerevisiae* (Abd1) with MTase buffer (50mM Tris-HCl, 40mM NaCl, 5mM DTT, 5mM EDTA) and 20 times more S-adenosylmethionine (SAM) than capG-RNA for 2 hours at 37°C. In order to obtain RNA with the cap1 structure, the purified recombinant vaccinia virus 2’-*O*-methyltransferase (VP39) was incubated with cap0-RNA in MTase buffer supplemented with 2mM MgCl_2_ and 20 times more SAM than RNA for 2 hours at 37°C. All differently capped RNAs were purified and quantitated as described above. Aliquots were digested with Nuclease P1 (NucP1) from *Penicillium citrinum* (Sigma-Aldrich, #N8630) for 2 hours at 37°C and run along with the standards ^m7^GpppG and GpppG on the same thin layer chromatography (TLC) plate (Sigma-Aldrich, #Z122882) in 0.45M (NH_4_)_2_SO_4_ buffer and visualised by UV shadowing to confirm the identity of the differently capped RNA substrates.

### Decapping activity

The decapping assay was performed by incubating 2μM of recombinant DXO with 1μM of capped RNA in IVDA-2 buffer (10mM Tris-HCl pH 7.5, 100mM KOAc, 2mM MgCl_2_, 0.5mM MnCl_2_, 2mM DTT, 0.2mM spermidine) at 37°C for 2 hours. Alternatively, each RNA substrate was treated with 0.5U NucP1 (Sigma-Aldrich, #N8630) in 100mM NaOAc pH 5.2. The reaction was stopped by adding formic acid to a final concentration of 500mM. Aliquots of the reactions (5CPM in lanes 1 and 3–12, 10CPM in lane 2) were applied to a polyethyleneimine-cellulose TLC plate (Sigma-Aldrich, #Z122882), which was developed with 0.3M (NH_4_)_2_SO_4_. The release of different cap products was revealed by autoradiography with a phosphorimager (Amersham Biosciences).

### Fluorescence spectroscopy

DXO intrinsic fluorescence was measured from 310nm to 440nm after excitation at 290nm using a Hitachi F-2500 fluorescence spectrophotometer. Background emission was eliminated by subtracting the signal from buffer alone. A maximal peak of tryptophan fluorescence emission was detected at 332nm.

The affinity of DXO for ^m7^GpppN-RNA (cap0) and ^m7^GpppN_m_-RNA (cap1) was determined by adding increasing concentrations of *in vitro* transcribed and capped RNA (0.46μM– 28μM) to a 1.2μM solution of DXO and monitoring fluorescence emission at 332nm. The binding studies were done in the absence of magnesium to prevent cleavage of the cap structure. Background emission was eliminated by subtracting the signal from buffer containing the appropriate amount of substrate. Data are shown as the mean ± SD of triplicates from two independent experiments.

The binding can be described by Eq (1):

$$K_{d}=\frac{\left[DXO\right]\left[ligand\right]}{\left[DXO∙ligand\right]}$$

where *K*_d_ is the apparent dissociation constant, [DXO] is the concentration of the protein, [DXO·ligand] is the concentration of complexed protein, and [ligand] is the concentration of unbound ligand in solution. The proportion of ligand-bound protein is related to the measured fluorescence emission intensity by Eq (2):

$$\Delta F/{\Delta F}_{max}=\frac{\left[DXO∙ligand\right]}{{\left[DXO\right]}_{tot}}$$

where ΔF is the magnitude of the difference between the observed fluorescence intensity at a given concentration of ligand and the fluorescence intensity in the absence of ligand, ΔF_max_ is the difference at infinite [ligand], and [DXO]_tot_ is the total protein concentration. If the total ligand concentration, [ligand]_tot_, is in large molar excess relative to [DXO]_tot_, then it can be assumed that [ligand] is approximately equal to [ligand]_tot_. Eqs (1) and (2) can then be combined to give Eq (3):

$$\Delta F/{\Delta F}_{max}=\frac{{\left[ligand\right]}_{tot}}{K_{d}+{\left[ligand\right]}_{tot}}$$

The K_d_ values were determined from a non-linear least-square regression analysis of titration data by using Eq (3).

### Exoribonuclease assay

Two 30-mer RNA oligonucleotides (^5’^GUAGUUCGCCUGUGUGAGCUGACAAACUUA^3’^) harbouring a chemically modified 2’-*O*-methyl nucleotide at either the first or the 16^th^ position from the 5’ end as well as a non-modified oligonucleotide were purchased from IDT. RNAs were labelled with [^32^P]pCp at their 3’ end by incubation with T4 ssRNA Ligase (NEB, #M0204S) for 1.5 hours at 37°C. RNA was precipitated with ethanol, resuspended in nanopure water and quantitated by spectrophotometry before being phosphorylated at the 5’ end by PNK kinase (NEB, #M0201S). RNA was purified by gel filtration using the Illustra Probe Quant G-50 micro columns (GE, #28-9034-08). The exoribonuclease assay was performed by incubating 2μM of recombinant DXO with 100nM of RNA in IVDA-2.1 buffer (10mM Tris-HCl pH 8.0, 5mM KOAc, 2mM MgCl_2_, 0.5mM MnCl_2_, 2mM DTT, 0.1mM spermidine) at 37°C for different time intervals. The reaction was stopped by the addition of EDTA to a final concentration of 100mM and reaction products were analyzed on a 20% polyacrylamide gel containing 8M urea. The radiolabeled RNA was visualized by autoradiography of the gel and was quantified with a phosphorimager (Amersham Biosciences).

### Immunofluorescence

The wild-type DXO gene as well as the K7A-R8A mutant was cloned into the pcDNA3.1+ mammalian expression vector. HeLa or HEK293T cells were seeded on coverslips (Fischer, #1254582) in a 24-well plate (20,000 cells per well) 24 hours before transfection and grown at 37°C and 5% CO_2_ in DMEM (Wisent, #319-015-CL) supplemented with 10% FBS (Wisent, #080–150) and 1mM sodium pyruvate (Wisent, #600-110-EL). Cells were transfected with pcDNA3.1+/DXO-WT and pcDNA3.1+/DXO-K7A-R8A using Lipofectamine 2000 transfection reagent (Thermo Fisher Scientific, #11668019) for 48 hours. The culture medium was removed, and the cells were washed in PBS prior to being fixed with a PBS solution containing 4% paraformaldehyde and 4% sucrose for 20min at room temperature (RT). Cells were washed with PBS before being permeabilized with 0.15% Triton X-100 in PBS for 5min at RT. Afterwards, cells were washed twice with PBS and blocked in 10% normal goat serum (NGS, Wisent, #053–150) for 20 minutes at RT. DXO was stained with a rabbit polyclonal DXO antibody (Proteintech, #11015-2-AP) diluted 1:100 in blocking solution (10% NGS) overnight at 4°C. Cells were washed three times with 10% NGS before being incubated in the dark for 1h at RT with an Alexa Fluor 488-labeled anti-rabbit secondary antibody (Cell Signaling, #4412) at a 1:1000 dilution in 10% NGS. All subsequent steps were carried out in the dark. Cells were washed three times with PBS at RT, and nuclei were stained with 1μg/ml Hoechst (Sigma-Aldrich, #861529) for 15 min at RT. After a final wash in PBS, the coverslips were mounted on slides using a SlowFade mounting medium (Thermo Fisher Scientific, #S36940). Epifluorescence microscopy was conducted using a Nikon TE2000E microscope. The fluorescent images were acquired at a 60x magnification and images were processed using the HCImage software.

## Results

### Decapping activity

**Fig 1:**
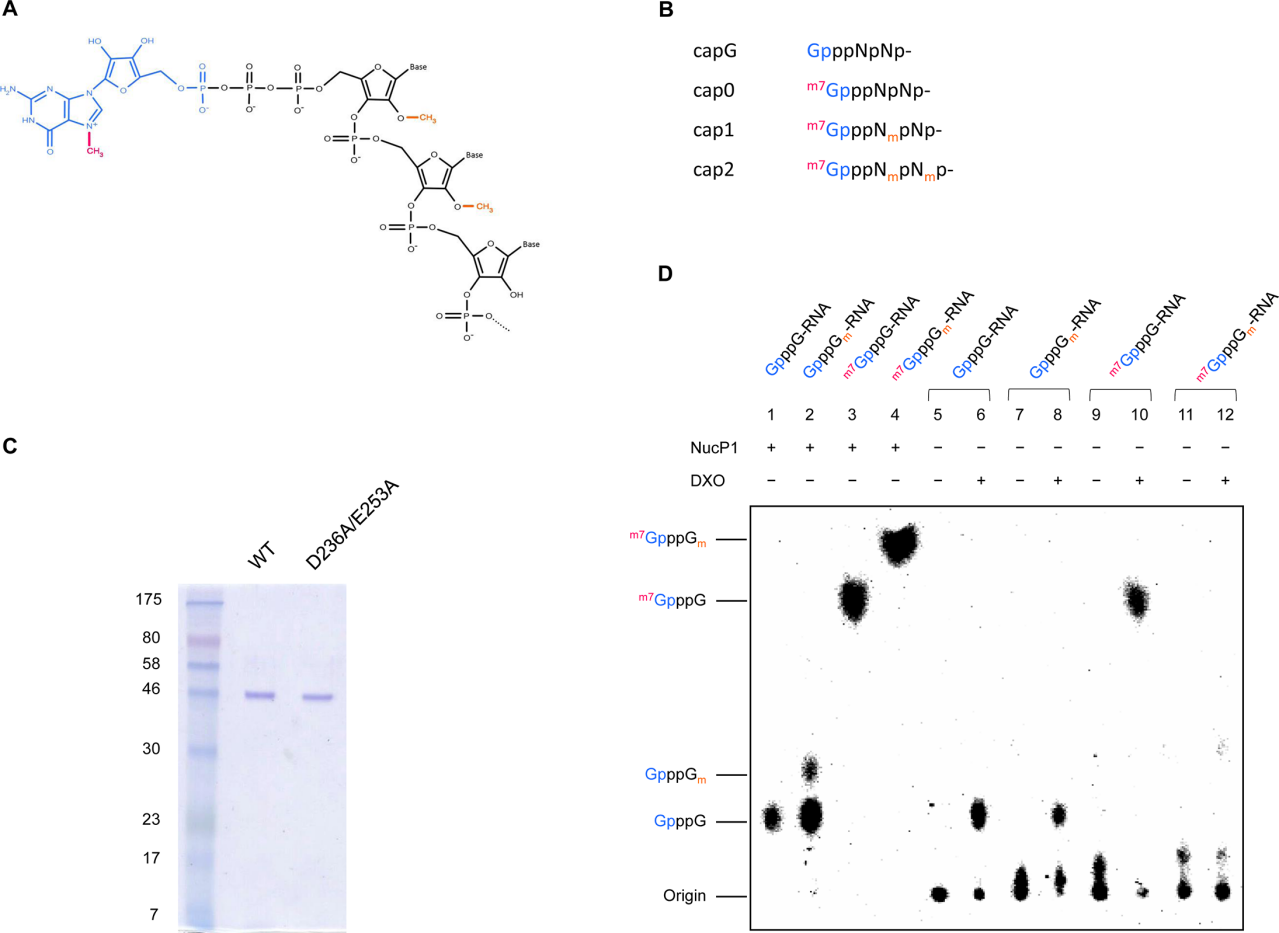
DXO hydrolyzes only capped RNAs without a 2’-*O*-methylation. (A) The RNA 5’ cap structure is composed of a guanosine (blue) linked to the RNA (black) through a 5’-5’ triphosphate bridge. The subsequent N7-methylation of the guanosine (magenta) confers a positive charge to the cap structure. Additional 2’-*O*-methylations (orange) can be found on the first few nucleotides. (B) Nomenclature of the different cap structures. (C) Aliquots (2μg) of the purified preparations of DXO and mutant DXO protein (D236A/E253A) were analyzed by electrophoresis through a 12.5% polyacrylamide gel containing 0.1% SDS and visualized with Coomassie Blue Dye. The positions and sizes (in kDa) of the size markers are indicated on the left. (D) RNAs harbouring different cap structures were transcribed and capped (incorporation of [α-^32^P]GTP) *in vitro*. They were then subjected to degradation by different enzymes, and reaction products were separated by thin layer chromatography. Lanes 1–4 show reaction products after treatment of differently capped RNAs with Nuclease P1. Degradation products after incubation of differently capped RNAs with purified DXO are shown in lanes 5–12. The origin of spotting and dinucleotide identities are listed on the left. NOTE: During the preparation of differently capped RNAs, only approximately 30% of GpppN-RNA was methylated to form GpppN_m_-RNA (lanes 2,7–8), resulting in a mixture of GpppN-RNA and GpppN_m_-RNA. Degradation products observed in lane 8 are due to the degradation of GpppN-RNA.

Since DXO has been identified as a key player in the mammalian mRNA cap quality control machinery [15], we decided to evaluate its decapping activity towards different physiological cap structures as well as a cap structure that contains the 2’-*O*-methylation but not the N7-methylation (Fig 1A and 1B). RNA substrates with cap0, capG, cap1 and cap2 structures were synthesized *in vitro* as described under 'Materials and methods'. During the synthesis of GpppG_m_-RNA, a methyl group was transferred to the 2’-*O* position of the first nucleotide of GpppG-RNA using the vaccinia virus 2’-*O*-methyltransferase (VP39). However, since non-N7-methylated RNA is not naturally found in mammalian cells, and thus constitutes an unusual substrate for the 2’-*O*-methyltransferase, only 30% of the substrate was successfully 2’-*O*-methylated, and the GpppG_m_-RNA substrate used in the decapping assay contains a significant amount of GpppG-RNA.

The human DXO protein was expressed in *E*. *coli* as described under 'Materials and methods' and purified by affinity chromatography on a Ni-NTA column. SDS-PAGE analysis showed that the 45.8-kDa DXO protein was the predominant polypeptide in the purified fraction (Fig 1C). The ability of DXO to decap the various RNA substrates was then investigated. The various radiolabelled RNA substrates were incubated with the enzyme and the reaction products were separated on polyethyleneimine-cellulose TLC plates and revealed by autoradiography. Nuclease P1, which catalyzes the hydrolysis of capped RNAs into single nucleotides, and a cap dinucleotide (GpppG) were used as controls, and the degradation products were run along the decapping reactions on the same TLC plate. As shown previously for mouse DXO [11], the human DXO protein is able to hydrolyze GpppN-RNA (capG) and ^m7^GpppN-RNA (cap0), to yield GpppN and ^m7^GpppN, respectively. However, DXO displayed no activity towards the ^m7^GpppN_m_-RNA substrate (cap1) (Fig 1D). We conclude that DXO can degrade incompletely capped RNAs (capG and cap0) while sparing fully capped RNAs (cap1).

Since RNAs harboring a cap0 structure (N7 methylation only) were decapped by DXO whereas RNAs with a cap1 structure (both N7 and 2’-*O*-methylations) were not, we tested the possibility that the sole presence of the 2’-*O*-methylation could prevent RNA degradation. RNAs were capped *in vitro*, and without the addition of a methylation at the N7 position, the substrates were subjected to 2’-*O*-methylation. Upon incubation with DXO, we observed that the enzyme was able to hydrolyze the capG substrate but failed to cleave the cap1 substrate (Fig 1D, lane 8). Therefore, the 2’-*O*-methylation is sufficient to prevent DXO decapping activity.

**Fig 2:**
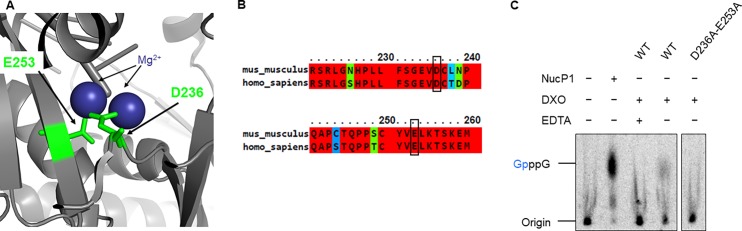
Catalytically inactive DXO. (A) Two residues involved in the coordination of Mg^2+^ ions in the active site of mouse DXO (pdb 4j7l) are shown. (B) Sequence conservation of residues coordinating Mg^2+^ ions between mouse and human DXO. (C) To ensure that the observed activity is indeed due to DXO, the catalytically inactive D236A/E253A mutant was used in a decapping assay using GpppG-RNA as substrate. Wild-type DXO readily hydrolyzes this cap structure in the presence of magnesium. However, no hydrolysis is observed with the catalytically inactive DXO mutant.

In order to ensure that the observed decapping activity is due to DXO, we intended to generate a catalytically inactive DXO mutant (D236A-E253A). Residue D236 was targeted since it was previously shown to coordinate an Mg^2+^ ion in the active site of the mouse DXO crystal structure (pdb 4j7l). Moreover, a mutation D236A in mouse DXO has previously been shown to abrogate its catalytic activity [10,15]. According to the crystal structure of mouse DXO (pdb 4j7l), residue E253 also contributes to the coordination of Mg^2+^ ions in the active site (Fig 2A). These two residues (D236 and E253) are conserved between mouse and human (Fig 2B). A D236A-E253A double mutant was therefore generated and used as a negative control in the decapping assay to confirm that the observed decapping activity is indeed due to DXO (Fig 1C). Only wild-type DXO showed decapping activity towards GpppG-RNA, thus confirming that the observed activity is due to DXO (Fig 2C, S1 Fig). Moreover, the addition of EDTA to the reaction confirmed the crucial role of magnesium in the reaction.

### Fluorescence spectroscopy

**Fig 3:**
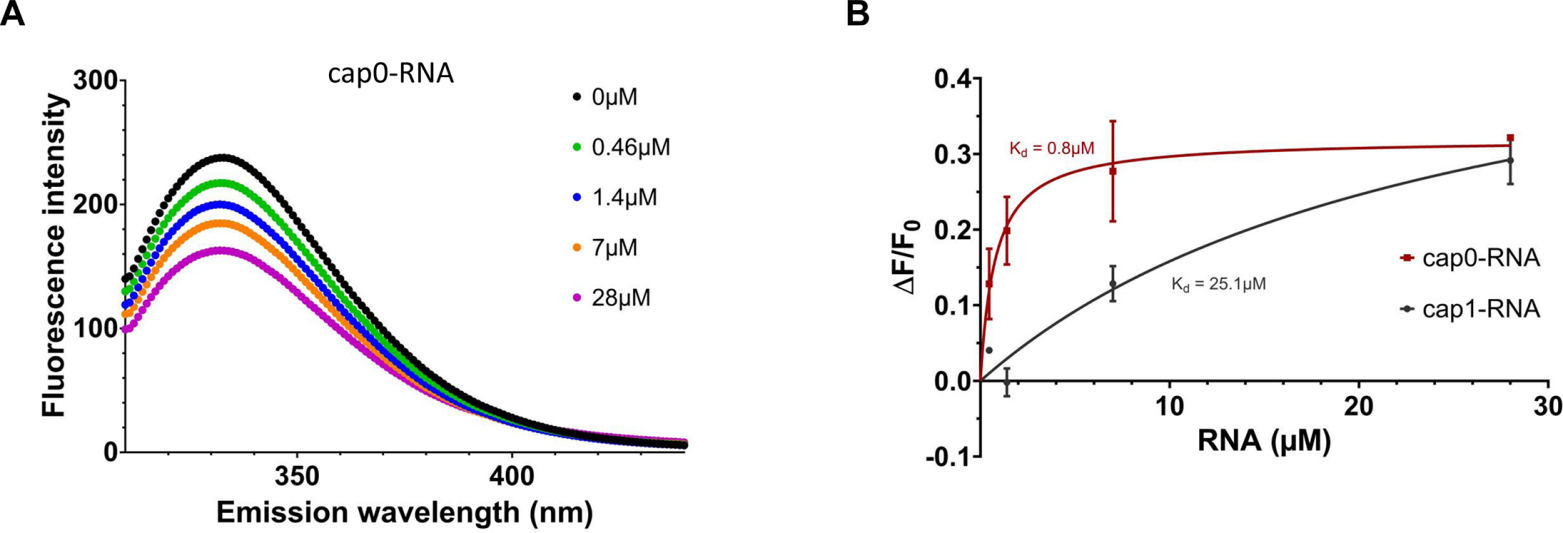
The presence of a 2’-*O*-methylation decreases affinity of DXO for capped RNA. The affinity of DXO for cap0-RNA and cap1-RNA were measured using fluorescence spectroscopy. (A) Increasing amounts of cap0-RNA (0.46μM– 28μM) were added to a of 1.2μM solution of DXO, and the emission spectrum was scanned from 310 to 440nm. (B) A saturation isotherm can be generated from these data by plotting the change in fluorescence intensity at 332nm as a function of added RNA. Results are shown as changes in intrinsic fluorescence in the presence of capped RNA compared to the absence of RNA (ΔF/F_0_).

Given that DXO does not hydrolyze cap structures that contain a 2’-*O*-methylation, we investigated its affinity for both ^m7^GpppG_m_-RNA (cap1-RNA) and ^m7^GpppG-RNA (cap0-RNA) using fluorescence spectroscopy. In the absence of RNA, a maximal peak of tryptophan fluorescence emission was detected at 332nm (Fig 3A). We observed that the binding of RNA to DXO resulted in an important modification of the intensity of the intrinsic fluorescence of the enzyme. As a consequence, we were able to evaluate the K_d_ value for RNA by titrating the binding of increasing amounts of RNA substrates to a fixed concentration of the enzyme. Typical emission spectra obtained from the titration of cap0-RNA are shown in Fig 3A. Increasing concentrations of cap0- and cap1-RNAs both led to a decrease in fluorescence emission at 332nm. The corresponding saturation isotherms generated by plotting the change in fluorescence intensity as a function of added substrates are shown in Fig 3B. About 30% of the intrinsic protein fluorescence was accessible to the quencher RNA substrate (Fig 3B). Our results indicate that the K_d_ for cap0-RNA (0.8μM) is approximately 30 times smaller than the K_d_ for cap1-RNA (25μM) indicating that the presence of the 2’-*O*-methylation on the cap structure drastically reduced the affinity of DXO for the RNA.

### Exoribonuclease activity

**Fig 4:**
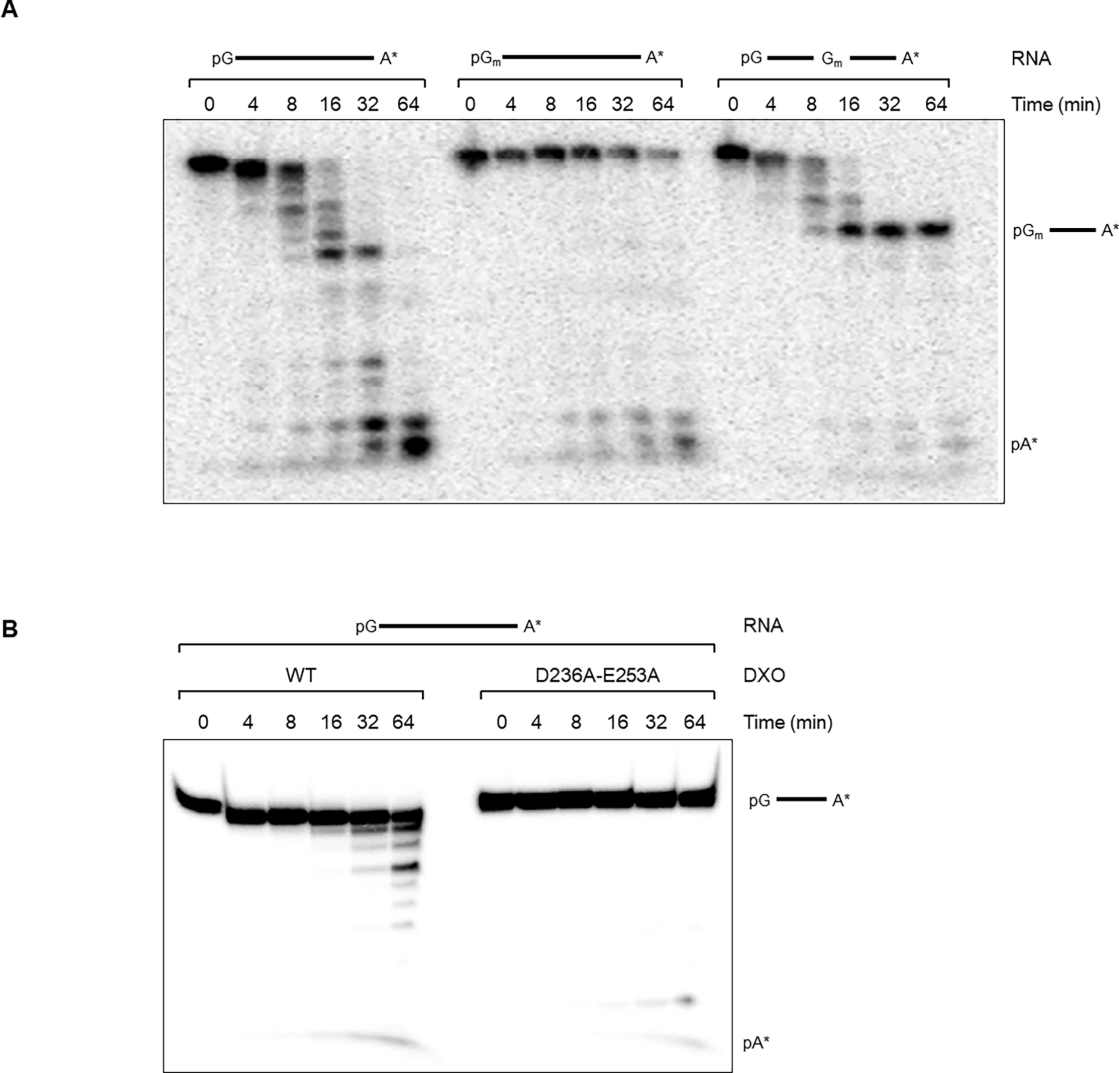
The presence of a 2’-*O*-methylation blocks the exoribonuclease activity of DXO. (A) The exoribonuclease activity of DXO toward different substrates was studied. 2μM of DXO were incubated with 100nM of the 30‐nt 3′‐radiolabelled RNA substrate harbouring either no 2’-*O*-methylation, a 2’-*O*-methylation on the first nucleotide or a 2’-*O*-methylation on the 16^th^ nucleotide. The reactions were incubated at 37°C for 0 to 64 minutes before being stopped by adding 100mM EDTA. Products were separated on a 20% denaturing polyacrylamide gel. (B) To ensure that the observed exoribonuclease activity is specific to the DXO protein, a catalytically inactive mutant (D236A-E253A) was used in an exoribonuclease assay with 5’ monophosphorylated RNA. Wild-type DXO readily degrades this RNA substrate, whereas almost no cleavage products are observed with the inactive mutant.

We next assessed the 5’-3’ exoribonuclease activity of DXO using a 5’-monophosphorylated 30-mer RNA oligonucleotide radiolabelled at its 3’ end. DXO showed time-dependent exoribonuclease activity towards a 5’ monophosphorylated RNA as observed by the formation of cleavage products (Fig 4A). Considering the inability of DXO to cleave the cap moiety on a 2’-*O*-methylated RNA (Fig 1D), we investigated the exonuclease activity of DXO towards an RNA substrate harboring a 2’-*O*-methylation on the first nucleotide. Using such a substrate, fewer degradation products were detectable, and they appeared at later times during the reaction. Since the presence of a methyl group on the first position was sufficient to prevent degradation by DXO, we investigated if the presence of the methyl group could block the exoribonuclease activity of DXO at other positions on RNAs. We used an RNA substrate modified with a single methyl group at the 2’-*O* position of the 16^th^ nucleotide. The pattern of degradation demonstrates that the enzyme degrades the RNA until it reaches the modified nucleotide (Fig 4A). We conclude that the presence of a 2’-*O*-methylated nucleotide prevents the DXO exoribonuclease activity.

To ensure that the observed exoribonuclease activity is indeed due to DXO, we used the catalytically inactive mutant D236A-E253A described above. When subjecting a 5’ monophosphorylated RNA substrate to either wild-type or mutant DXO, degradation products are only observed in the presence of the wild-type protein, thus confirming that the observed activity is due to DXO (Fig 4B).

### DXO cellular localization & identification of a functional NLS

**Fig 5:**
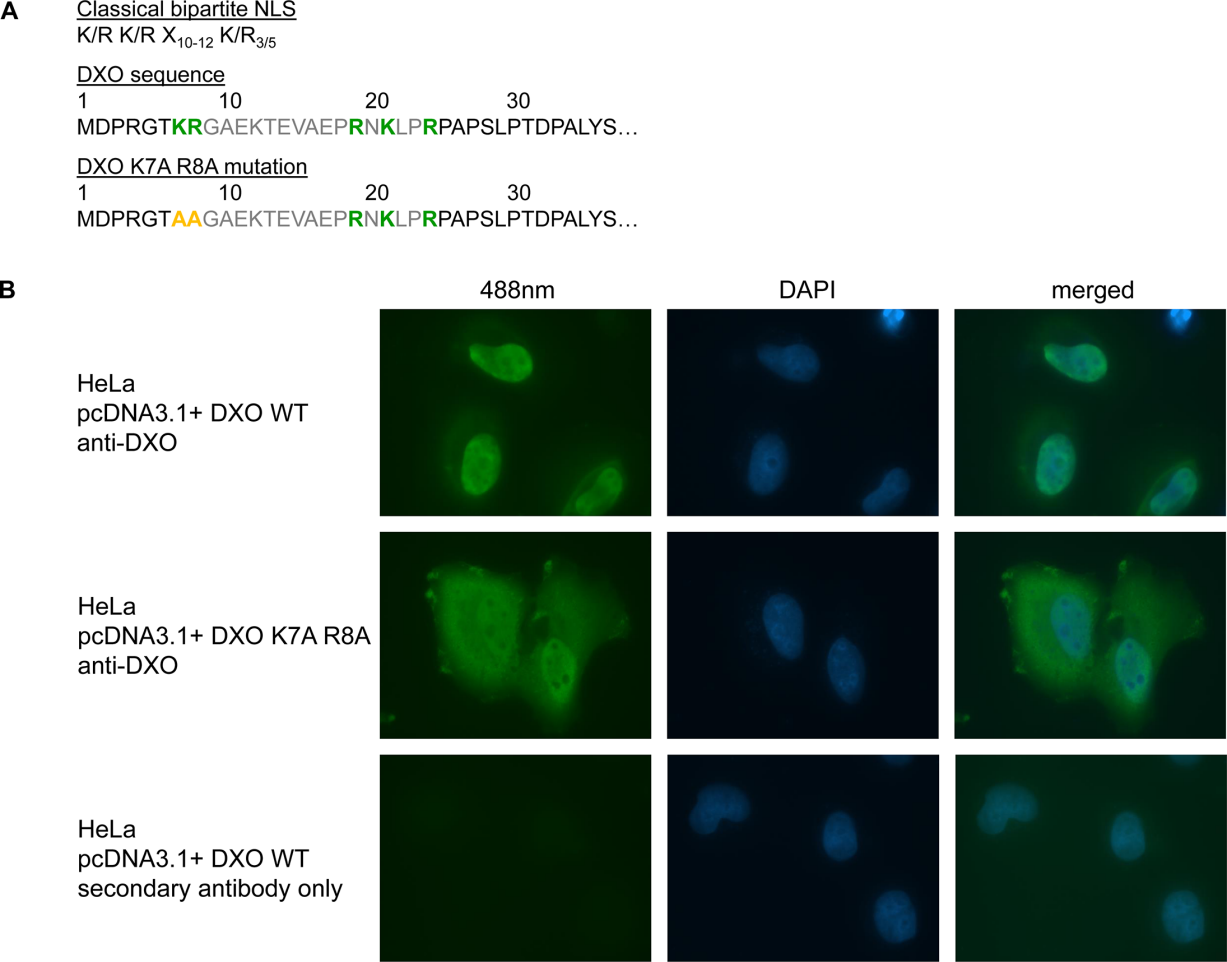
DXO contains a functional NLS as shown by site-directed mutagenesis and immunofluorescence. (A) A schematic representation of a classical bipartite NLS consensus sequence and the corresponding sequence at the N-terminal end of DXO. (B) HeLa cells were transfected with pcDNA3.1+/DXO and pcDNA3.1+/DXO-K7A-R8A (mutations in the NLS) using Lipofectamine 2000. DXO localization was monitored 48 hours post-transfection by immunofluorescence using a rabbit polyclonal DXO antibody and a polyclonal anti-rabbit antibody coupled to Alexa Fluor 488. Fluorescent images were gathered using an epifluorescence microscope with a 60x objective. Exposure times were identical in all three conditions.

The proposed DXO function in an mRNA cap quality control mechanism would likely require a nuclear localization. Indeed, the nuclear localization of the mouse DXO was previously shown to be nuclear [17]. Close inspection of the amino-terminus region of DXO revealed the presence of amino acid stretches rich in lysine and arginine residues (Fig 5A). These clusters of basic residues present similarities with a bipartite nuclear localisation signal (NLS) which is typically defined as two basic clusters separated by a spacer region of any 10 amino acids. Residues K7 and R8 of human DXO constitute the first cluster of basic amino acids of the presumed NLS. We substituted these two residues by alanine residues and determined the impact on the cellular localization of the mutant by immunofluorescence. As expected, the wild-type DXO protein is present predominantly within the nucleus. However, the DXO K7A-R8A mutant is located in both the nucleus and in the cytoplasm (Fig 5B), indicating that the identified sequence at the amino terminus of DXO is indeed a functional nuclear localisation signal.

## Discussion

In recent years, various studies have demonstrated the presence of aberrant cellular pre-mRNAs which are incompletely capped [11,14]. However, difficulties have been encountered in the identification of the enzymes involved in the degradation of these aberrant transcripts since defective RNA species could not serve as substrates for the classical decapping enzymes, such as Dcp2 and Nudt16, which are specific for the fully capped transcripts [18]. However, progress has been made with the recent identification of the DXO protein which can both decap and degrade RNA transcripts with aberrant 5’ ends such as pRNA, pppRNA, GpppRNA (capG) and, surprisingly, ^m7^GpppRNA (cap0) [15]. An hypothesis was suggested in which the mammalian quality control mechanism would lead to the degradation of all incompletely capped mRNAs by DXO, independently of the nature of their 5’ end, unless they were initially bound by cap binding complex. However, the importance of the 2'-*O*-methylation of the first transcribed nucleotide was never investigated in this context. In the present study, we demonstrate that DXO senses and spares fully capped (cap1) RNA transcripts while selectively degrading transcripts lacking 2’-*O*-methylation (such as cap0 and capG). These results are consistent with a quality control mechanism that degrades incompletely capped RNAs (capG and cap0) while sparing fully capped RNAs (cap1).

The present study showed that RNAs bearing immature cap structures, such as Gppp-RNA and ^m7^Gppp-RNA, were decapped by DXO while ^m7^GpppN_m_-RNA was resistant to its activity. In this context, the 2’-*O*-methylation appears to be the key element defining mRNA as fully capped and suitable for survival, export and translation. These results are further supported by the observation that DXO affinity for RNA is drastically reduced by the presence of 2’-*O*-methylation on the RNA substrate. Moreover, the DXO 5’-3’ exoribonuclease activity is blocked by the presence of a 2’-*O*-methylation on the RNA substrate. These findings suggest that DXO-mediated mRNA quality control is dependent on the 2’-*O*-methylation status of pre-mRNAs rather than on the competition of CBC and DXO for mRNA binding as proposed previously [15].

The human DXO protein appears as a key component of the mammalian RNA decapping pathway. Mounting evidence suggest that RNA decapping is a highly regulated process which involves both positive and negative regulators. In mammalian cells, Hedls was shown to be a positive effector of Dcp2 decapping [19]. The eukaryotic initiation factor 4A (eIF4A) is another modulator of RNA decapping which can inhibit decapping in vitro [20], most likely by binding to the 5' cap thereby preventing access to the decapping enzyme. Finally, the VCX-A protein, which has been implicated in X-linked mental retardation, has been shown to preferentially associate with the 5' cap and inhibit decapping [21,22]. A two-hybrid screening has previously predicted interactions between DXO and the cytoplasmic Xrn1 and nuclear Xrn2, two exoribonucleases involved in the degradation of diverse RNA substrates during general RNA decay [23]. The ability of additional cellular factors to interact with DXO and modulate its activity remains to be investigated.

**Fig 6:**
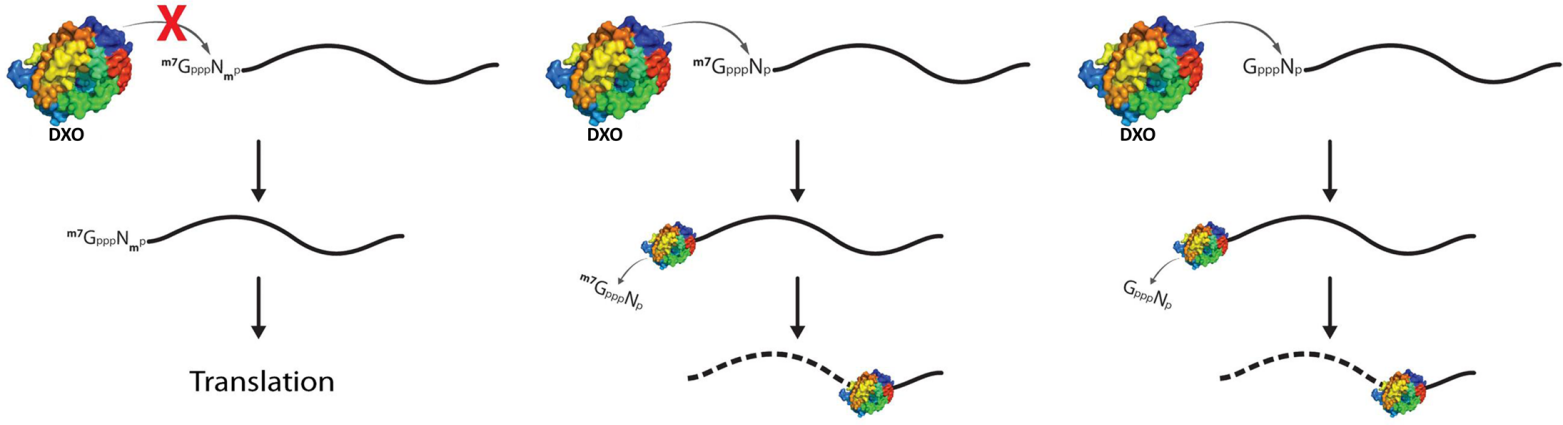
Model of DXO activity on RNA. DXO removes incomplete cap structures such as capG (right) and cap0 (middle) and degrades the resulting uncapped mRNA, whereas RNAs harboring a cap1 structure (left) are unaffected. RNAs with capG and cap0 structures can be either capping intermediates or non-self RNAs.

Overall, we have shown that DXO is a multifunctional enzyme that possesses both decapping and 5’-3’ exoribonuclease activities toward non-2’-*O*-methylated RNA transcripts. DXO is directly involved in the mRNA quality control mechanism by degrading mRNAs with abnormal cap structures (Fig 6). It is tempting to speculate that the elimination of these aberrant mRNAs is essential to avoid their export to the cytoplasm and recognition as extrinsic RNAs, which could potentially lead to the activation of the innate immune response. Additional studies are clearly needed to confirm the role, if any, of DXO in this process.

## Supporting information

S1 Fig Decapping assay with DXO mutants. Three mutant DXO proteins were generated and tested in a decapping assay using GpppG-RNA as substrate. Wild-type DXO as well as the R145A-Q146A-E147A and R177A-M185A mutants readily cleave this cap structure. However, no hydrolysis is observed with the D236A-E253A mutant. This figure shows the original, uncropped image that has been used to generate Fig 2C.
(TIF)
