# Correction: 2'-*O*-methylation of the mRNA cap protects RNAs from decapping and degradation by DXO

**DOI:** 10.1371/journal.pone.0202308

**Published:** 2018-08-09

**Authors:** Frédéric Picard-Jean, Carolin Brand, Maude Tremblay-Létourneau, Andréa Allaire, Maxime C. Beaudoin, Simon Boudreault, Cyntia Duval, Julien Rainville-Sirois, Francis Robert, Jerry Pelletier, Brian J. Geiss, Martin Bisaillon

The Data Availability statement for this paper is incorrect. The correct statement is: The authors confirm that all data underlying the findings are fully available without restriction. All data underlying the study are within the paper and its Supporting Information files.

Fig 1 is incorrect. The authors have provided a corrected version here.

**Fig 1:**
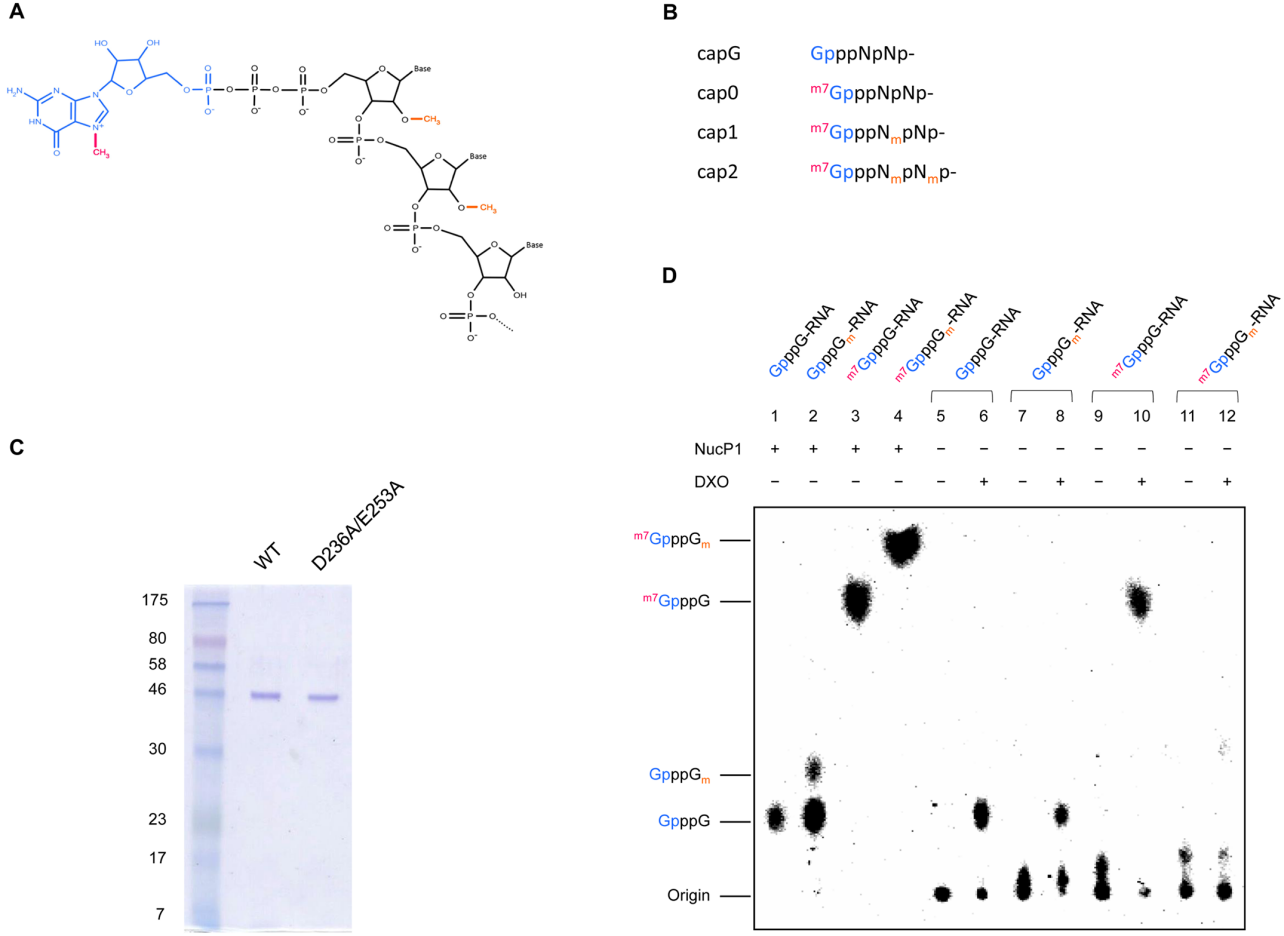
DXO hydrolyzes only capped RNAs without a 2’-*O*-methylation. (A) The RNA 5’ cap structure is composed of a guanosine (blue) linked to the RNA (black) through a 5’-5’ triphosphate bridge. The subsequent N7-methylation of the guanosine (magenta) confers a positive charge to the cap structure. Additional 2’-*O*-methylations (orange) can be found on the first few nucleotides. (B) Nomenclature of the different cap structures. (C) Aliquots (2μg) of the purified preparations of DXO and mutant DXO protein (D236A/E253A) were analyzed by electrophoresis through a 12.5% polyacrylamide gel containing 0.1% SDS and visualized with Coomassie Blue Dye. The positions and sizes (in kDa) of the size markers are indicated on the left. (D) RNAs harbouring different cap structures were transcribed and capped (incorporation of [α-^32^P]GTP) *in vitro*. They were then subjected to degradation by different enzymes, and reaction products were separated by thin layer chromatography. Lanes 1–4 show reaction products after treatment of differently capped RNAs with Nuclease P1. Degradation products after incubation of differently capped RNAs with purified DXO are shown in lanes 5–12. The origin of spotting and dinucleotide identities are listed on the left. NOTE: During the preparation of differently capped RNAs, only approximately 30% of GpppN-RNA was methylated to form GpppN_m_-RNA (lanes 2,7–8), resulting in a mixture of GpppN-RNA and GpppN_m_-RNA. Degradation products observed in lane 8 are due to the degradation of GpppN-RNA.

## References

[1] Picard-Jean F, Brand C, Tremblay-Létourneau M, Allaire A, Beaudoin MC, Boudreault S, et al (2018) 2'-*O*-methylation of the mRNA cap protects RNAs from decapping and degradation by DXO. PLoS ONE 13(3): e0193804 10.1371/journal.pone.0193804 29601584 PMC5877831

