# Supplementary figures and images for: 2'-O-methylation of the mRNA cap protects RNAs from decapping and degradation by DXO
Source: PLoS One. 2018 Mar 30;13(3):e0193804. doi: 10.1371/journal.pone.0193804 (PMC5877831; doi:10.1371/journal.pone.0193804)

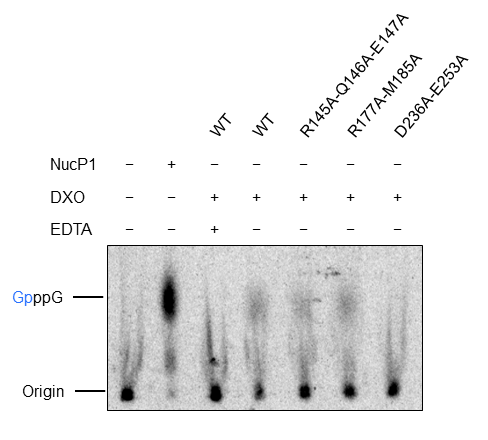

Supplement: S1 Fig — Three mutant DXO proteins were generated and tested in a decapping assay using GpppG-RNA as substrate. Wild-type DXO as well as the R145A-Q146A-E147A and R177A-M185A mutants readily cleave this cap structure. However, no hydrolysis is observed with the D236A-E253A mutant. This figure shows the original, uncropped image that has been used to generate Fig 2C. (TIF) [file pone.0193804.s001.tif]
